# Modelling the roles of antibody titre and avidity in protection from *Plasmodium falciparum* malaria infection following RTS,S/AS01 vaccination

**DOI:** 10.1016/j.vaccine.2020.09.069

**Published:** 2020-11-03

**Authors:** Hayley A. Thompson, Alexandra B. Hogan, Patrick G.T. Walker, Michael T. White, Aubrey J. Cunnington, Christian F. Ockenhouse, Azra C. Ghani

**Affiliations:** a MRC Centre for Global Infectious Disease Analysis, Department of Infectious Disease Epidemiology, Imperial College London, London, United Kingdom; b Malaria: Parasites and Hosts, Department of Parasites and Insect Vectors, Institut Pasteur, Paris, France; c Section of Paediatrics, Imperial College London, London, United Kingdom; d PATH Malaria Vaccine Initiative, Washington, DC, United States

**Keywords:** Malaria, Sporozoites, Antibody response, Correlates of protection, Mathematical modelling, Predictive vaccine efficacy

## Abstract

Anti-circumsporozoite antibody titres have been established as an essential indicator for evaluating the immunogenicity and protective capacity of the RTS,S/AS01 malaria vaccine. However, a new delayed-fractional dose regime of the vaccine was recently shown to increase vaccine efficacy, from 62.5% (95% CI 29.4–80.1%) under the original dosing schedule to 86.7% (95% CI, 66.8–94.6%) without a corresponding increase in antibody titres. Here we reanalyse the antibody data from this challenge trial to determine whether IgG avidity may help to explain efficacy better than IgG titre alone by adapting a within-host mathematical model of sporozoite inoculation. We demonstrate that a model incorporating titre and avidity provides a substantially better fit to the data than titre alone. These results also suggest that in individuals with a high antibody titre response that also show high avidity (both metrics in the top tercile of observed values) delayed-fractional vaccination provided near perfect protection upon first challenge (98.2% [95% Credible Interval 91.6–99.7%]). This finding suggests that the quality of the vaccine induced antibody response is likely to be an important determinant in the development of highly efficacious pre-erythrocytic vaccines against malaria.

## Highlights

• Models capturing key malaria life-cycle stages can help us evaluate vaccine candidates.
• Model fitting revealed antibody avidity to be an important determinant of RTS,S vaccine efficacy.
• High avidity and titre were associated with increased levels of vaccine efficacy.
• Did not identify any thresholds of protection for either immune marker.

## 1 Introduction

Despite unprecedented success in reducing the burden of malaria over the last decade, the disease remains a major global health problem. An estimated 228 million cases and 405,000 deaths from malaria occurred in 2018 alone, over 90% of which were caused by *Plasmodium falciparum* [1]*.* Progress in reducing cases and deaths from malaria has stalled in recent years and developing a highly efficacious and durable protective childhood malaria vaccine therefore remains a research priority.

The first malaria vaccine to complete Phase III field trials, RTS,S/AS01, has begun large scale pilot implementation in three sites across Sub-Saharan Africa [2]. RTS,S is a pre-erythrocytic recombinant sub-unit vaccine that fuses a portion of the *P. falciparum* circumsporozoite protein (CSP) and the hepatitis B surface antigen and is delivered with a potent adjuvant system AS01 [3], [4]. It includes both B cell and T cell epitopes of CSP: the central repeat region (NANP) and portions of the C-terminal nonrepeat region [3]. As a pre-erythrocytic vaccine, RTS,S aims to induce an immune response that prevents sporozoites traversing the peripheral blood circulation and completing liver stage development [5], [6]. RTS,S/AS01 is the only vaccine against malaria that has demonstrated partially protective efficacy in children across a range of transmission settings in Sub-Saharan Africa [7]. With efficacy reaching 36.3% (95% CI 31.8%–40.5%) in children aged 5–17 months at first vaccination, when given on a three dose monthly schedule with a fourth dose 18 months after dose three. Efficacy was demonstrated to wane significantly over the course of follow up [7], [8].

To reach the World Health Organization Malaria Vaccine Technology Roadmap target of a protective malaria vaccine of 75% efficacy over two years, further development of the RTS,S vaccine and other next-generation platforms will be required [9]. However, further optimisation is challenging as the definitive mechanisms by which RTS,S confers protection are not fully understood and immune correlates of protection not fully defined. RTS,S has been shown to be highly immunogenic, consistently inducing high titres of CSP-specific antibodies and high numbers of CD4+ T cells in malaria naïve individuals and in naturally exposed populations [10], [11], [12], [13], [14], [15], [16], [17]. Anti-NANP antibody titres have been established as a major correlate of protection, with higher titres associated with protection against infection and the rate of waning of antibody responses following vaccination associated with the magnitude and duration of efficacy over time [10], [18], [19]. However, there remains significant residual variability in protection that is unexplained after accounting for antibody titre and CD4^+^ T cell counts [10], [11], [12], [13], [14], [15], [16], [17] and no clear protective thresholds for immune markers have been identified [20].

Controlled Human Malaria Infection Challenge Studies facilitate the early evaluation of pre-erythrocytic vaccines and enable detailed immunological assessments to be undertaken in malaria naïve individuals [21], [22]. These studies offer a unique opportunity to study immune mechanisms and correlates of protection following vaccination and timed parasite exposure. Recent work by Regules et al. in the optimization of RTS,S indicated that greater initial vaccine efficacy could be achieved through an alteration to the vaccination schedule and dose amount, whereby the third dose of RTS,S was given with a 5-month delay and at one-fifth of the standard dose (Fx017M) [23]. Efficacy against clinical disease was 86.7% (95% CI 66.8–94.6%) for those volunteers in the delayed-fractional arm at first challenge compared to 62.5% (95% CI 29.4–80.1%) for volunteers on the standard 0, 1, 2-month full dose regime (012M) [23]. The immunological reasons for the difference in efficacy are not fully understood, and the increase in efficacy did not correlate with increases in antibody titre. Instead, it was postulated that the improvement in efficacy resulted from an increase in affinity maturation resulting in a higher quality of antibody response following the delayed-fractional schedule [23]. Affinity maturation results in mutated antibody variants with improved antigen-binding properties to better protect from invading pathogens [24].

Given the variation in protection that remains unexplained by anti-CSP antibody titre alone, the quality of antibodies has continued to be theorised to contribute to vaccine efficacy [6], [25]. The quality of antibodies can be eluded to by taking measurements of avidity. Antibody avidity measured using inhibition ELISAs is a representation of the overall strength of interaction between antibodies and antigens in a complex, it takes into account the intrinsic affinity of antibodies to their specific epitopes and also valences of antibodies and any structural features of antibody binding confirmations [26], [27], [28]. High avidity antibodies have been shown to be important in the protection conferred by several viral and bacterial vaccines [29], [30], [31], [32], [33] but studies of antibody avidity responses following 012M RTS,S vaccination during field trials have provided conflicting evidence on the relationship between avidity and protection from infection thus far [34], [35], [36], [37].

Here we re-analyse the immunological data from the delayed-fractional challenge trial using a biologically-motivated mathematical model of *P. falciparum* sporozoite inoculation [10]. This approach allows us to combine data on the relative risk of infection and the time to onset of detectable parasitaemia in those infected into a single measure of vaccine efficacy. We extend this model to include avidity indices to determine the relative contributions of anti-NANP IgG titre and avidity to vaccine-induced protection. And aim to determine if avidity is a potential correlate of vaccine induced protection in RTS,S vaccinated populations. While changes in avidity were postulated to have a potentially causal role in the difference between vaccine efficacy between the two arms in the challenge study, the link between avidity and individual level protection has not yet been quantified. The use of modelled dose–response curves in this study enables us to relate the magnitude of the immune response to sporozoite survival probability providing insights into the likely contribution of these antibody characteristics in driving RTS,S efficacy.

## 2 Methods

### 2.1 Challenge trial data

Data were obtained from a Phase 2a RTS,S/AS01 challenge study, detailed trial descriptions can be found in in Regules et al. [23]. Briefly, 46 malaria naïve adults, defined as those with no previous history of malaria or receipt of an investigational malaria vaccine (mean age of 33.6 years), received full vaccination schedules, 30 on the Fx017M schedule and 16 on the 012M schedule. Volunteers underwent mosquito challenge with the bites of five *P. falciparum* (3D7, a clone of the NF54 strain) infected *Anopheles stephensi* mosquitoes three weeks after the third vaccine dose. Parasitemia levels were monitored through blood slide reading and PCR with monitoring performed daily from days 5–20 after challenge and then every two days thereafter up to 28 days post-challenge. Volunteers who tested positive for malaria during the follow-up period were treated with chloroquine phosphate. From the 46 volunteers who underwent vaccination and challenge time to onset of parasitemia and measurements of vaccine-induced immune responses were recorded.

Immunogenicity assessments are described in detail in Regules et al. [23]. Briefly, IgG antibody levels were measured for several regions of the CSP protein, here we focus on the NANP repeat region. Measurements were made by standard enzyme linked immunosorbent assays (ELISAs) [38], using plate absorbed R32LR antigen, with total IgG titre reported in ELISA Units as per the Walter Reed Army Institute of Research assay. ELISA-based avidity assays were conducted to assess antibody binding to the repeat region NANP. These findings were reported as an avidity index calculated by dividing the serum titre in the presence of 4M urea (the chaotropic reagent) to the serum titre obtained without exposure to the chaotropic agent. For the following analysis we used measurements of end-point total IgG titre and avidity indices against the NANP repeat region taken from a single time point in each volunteer on the day of mosquito challenge.

### 2.2 Statistical methods

Immunological data was re-evaluated to assess for any statistically significant difference in immune measurements. Non-parametric Mann-Whitney U tests were used to compare antibody titres and avidity indices between vaccination schedule arms. A Spearman’s rho was calculated to test for any correlation between immune measurements and a log rank test was performed to test for significant differences in time to onset of parasitaemia. Fishers exact test was used to compare the observed efficacy in the delayed-fractional vs the standard arms.

### 2.3 Mathematical model

**Fig. 1:**
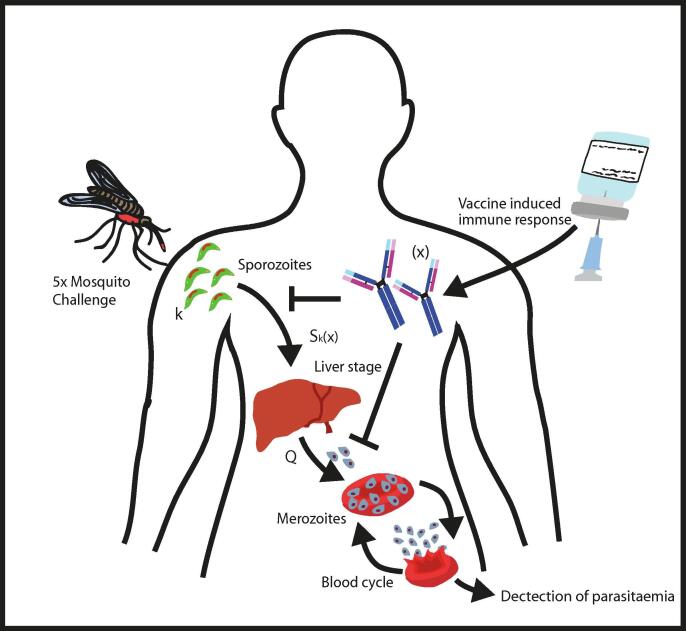
**Sporozoite Infection Model.** The sporozoite infection model mathematically captures the parasite and immune dynamics following vaccination and challenge. This schematic conceptualises the model: following mosquito challenge with 5 infectious mosquitos, inoculated sporozoites *(k)* migrate to the liver, undergo intrahepatic development, and release an initial infectious dose of merozoites *(Q)*. These merozoites cycle through blood stage development until they reach a threshold level for detection. Following vaccination with RTS,S the number of antibodies and the avidity of antibodies (*x*) will influence the survival probability of the inoculated sporozoites such that the probability that sporozoites will successfully initiate blood stage infection, *S_k_*, will become reduced as function of the immune response: *S_k_*(*x*). In addition, the antibody immune response will also influence the initial infectious dose of merozoites (*Q*), if the number of sporozoites that successfully initiate liver stage infection are reduced so too will the initial load of merozoites emerging from the liver which can be related back to longer delays in detection of parasitaemia. If an individual is protected, then we assume all infectious sporozoites will have been prevented from completing liver stage development.

We used the sporozoite infection model previously described in White et al. [10]. The model is an individual based biologically-motivated mathematical model that captures *P. falciparum* parasite dynamics following inoculation. We assume that following vaccination with RTS,S the antibody response is the main driver of protection. The model (Fig. 1) characterises mathematically the relationships between the number of sporozoites inoculated following challenge (k) and the production of merozoite progeny (Q) that leads to detectable infection. Briefly, mosquitoes inject sporozoites into volunteers, these sporozoites will then go on to invade liver cells with a certain probability ($S_{k}$) and produce merozoite progeny which once released into the blood may proceed to cause clinical disease. Following vaccination with RTS,S the quantity and quality of antibodies (x) will influence the survival probability of the inoculated sporozoites such that the probability that sporozoites will successfully initiate blood stage infection, $S_{k}$, will become reduced as function of the immune response: $S_{k}\left(x\right).$ We assume that if a volunteer is protected, then none of the inoculated sporozoites will be successful in producing merozoite progeny and infection will be prevented. If an individual becomes infected, then infection will have been initiated by some number of sporozoites which then release a given number of merozoites into the blood stream*.* A reduction in the number of successful sporozoites as a result of vaccine induced immune responses will cause a reduced liver to blood inocula (Q), leading to a longer time to onset of detectable parasitaemia. This delay in the onset of parasitaemia can be used to calculate the number of merozoites that initiate blood-stage infection as: $Q=p_{T}m^{-(T-T_{L})}$. Where $t_{L}$ = 6.5 days needed for intrahepatic development [39], [40], $P_{T}$ the threshold number of merozoites needed for detection of infection by microscopy (50,000,000 parasites) [41] and m the fixed daily blood stage replication rate (3.8 day^−1^) [42].

To quantify the contribution of the antibody response to protection status we adapted the model and incorporated two dose–response curves that relate IgG titre and avidity indices (dose) to the probability that a sporozoite survives the given immune response (response). Two parametric dose–response curves were considered when characterising these relationships: exponential and Hill functions. For an exponential dose–response, the probability that a sporozoite survives immune response x of a given magnitude is: ${f\left(x\right)=e}^{-log\left(2\right)\frac{x}{\beta }}$. For a Hill function the probability is given by: $f\left(x\right)=\frac{1}{1+{\left(\frac{x}{\beta }\right)}^{\alpha }}$ where α and β are the shape and scale parameters of the respective dose–response curves to be estimated during model fitting (See Supplementary Information for further descriptions).

We used this model to estimate several key biological parameters: the mean (n) and standard deviation $\left(\sigma_{n}\right)$ in the number of successful sporozoites inoculated per challenge and the resulting standard deviation in the number of merozoite progeny per sporozoite $\left(\sigma_{\mu }\right)$ (Table 1). The sporozoite infection model in this form thus allows us to utilise the data available in the binary outcome of infection or protection, and the data in time to onset of parasitemia in infected volunteers. An alternative approach is detailed in the Supplementary Methods where reductions in parasite load are ignored (and thus information on delays in the time to detection of parasitaemia) and the binary outcome of protection or infection is used to fit the model. In this instance we cannot model a dose–response relationship between the antibody markers and sporozoite survival but instead between the antibody response and protection from infection directly.

**Table 1:** **Parameters describing the biology of *P. falciparum* infection.** Estimated parameters from the best fit Hill-Exponential dose–response model are dependent on the fixed parameter values for the duration of liver stage development and daily blood stage multiplication rate and average number of merozoites released per sporozoite.

**Parameter**	**Description**	**MCMC estimate**	**95% Credible Interval**
**n**	Mean number of successful sporozoites per challenge	134	72–242
**σ_n_**	Standard deviation of the number of sporozoites per challenge	177	92–304
**σ_µ_**	Standard deviation in the number of merozoites per sporozoite	71,427	56,570–91,287
**β_ab_**	Anti-NANP antibody titre needed for 50% reduction in sporozoite survival probability	8,639	2,881–20,528
**α_ab_**	Shape parameter for antibody dose–response	1.53	0.36–3.71
**β_av_**	Antibody avidity index needed for 50% reduction in sporozoite survival probability	7.92	6.0–12.31

**Fixed Parameters**			**Reference**

**t_L_**	Duration of liver stage development	6.5 days	[39], [40]
**m**	Daily blood stage parasite multiplication rate	3.8 day^−1^	[42]
**P_T_**	Threshold number of parasites for detection of infection (occurrence of parasitaemia, defined by positive blood slide)	50,000,000 parasites	[41]
**µ**	Mean number of merozoites released per sporozoite	30,000	[40]

### 2.4 Model fitting

Model parameters were jointly estimated in a Bayesian framework by fitting to data (immunological measurements, infection status and time to onset of parasitaemia) from both vaccination arms. Using Metropolis-Hastings techniques, we sampled from the joint posterior distribution of parameter values applying a Robbins-Munro algorithm for adaptive tuning of the proposal distribution to ensure good mixing. The priors for several key parameters were informed using the published studies by White et al. [10] and Coffeng et al. [40] (Table S2). Model parameter estimates were summarised in terms of the median and 95% Bayesian credible intervals (95% CrI) of the posterior samples for each parameter (Table 1). We compared the fit of models incorporating avidity-dependent parasite mortality, along with those where efficacy was solely dependent upon titre using the Deviance Information Criterion (DIC).

### 2.5 Predictive vaccine efficacy

Following model fitting, parameters from the best fitting model were used to obtain estimates of vaccine efficacy. For an infection to be prevented, an individual’s vaccine-induced immune response must act to kill all invading sporozoites. Vaccine efficacy against infection $\left({\mathrm{VE}}_{i}\right)$ was defined as the reduction in the probability of infection following infectious challenge in vaccinated volunteers compared to control volunteers [10]. As we assumed that the number of successful sporozoites follows a Negative Binomial distribution. Efficacy against infection was estimated as:

$${\mathrm{VE}}_{i}\left(x\right)=1-\frac{1-{\left(\frac{r}{nf\left(x\right)+r}\right)}^{r}}{1-{\left(\frac{r}{n+r}\right)}^{r}},$$

where x denotes the potential combination of titre and avidity measurements, n is the mean number of successful sporozoites multiplied by the sporozoite survival probability f(x) and r the shape parameter of the negative binomial distribution: $r=\frac{n^{2}}{\sigma_{n-n}^{2}}$. Vaccine efficacy per sporozoite $\left({\mathrm{VE}}_{s}\right)$ was defined as the proportional reduction in liver-stage parasite load [10] (the liver-blood inoculum) and was calculated as: ${\mathrm{VE}}_{s}\left(x\right)=1-f\left(x\right)$.

### 2.6 Predictive time to onset of parasitaemia

From our model calculations of the liver-to-blood parasite inocula from each volunteer Q(x), we estimated expected time to onset of parasitemia given their immune response (x) as:

$$T\left(x\right)=t_{L}+\frac{\log\left(\frac{P}{Q(x)}\right)}{log(m)}$$

## 3 Results

### 3.1 Immunogenicity data

Of the 30 volunteers receiving the delayed-fractional schedule 4/30 became infected after first challenge, three weeks post dose three, (Vaccine Efficacy: 86.7% [95% CI 66.8–94.6%]) and 6/16 became infected following the standard schedule (Vaccine Efficacy: 62.5% [95% CI 29.4–80.1%]) [23]. This difference was not-significant when assessed using the Fisher-exact test (p-value = 0.074). Despite the observed increase in protective efficacy of the delayed-fractional regime, anti-NANP antibody levels were lower in the subjects receiving this regime than in those receiving the standard regime on the day of challenge (Mann-Whitney: p-value = 0.02). Whereas anti-NANP antibody avidity was significantly higher in volunteers from the delayed fractional arm (Mann-Whitney: p-value = 0.03). Furthermore, there was a trend for protected volunteers to achieve higher avidity and titre measurements within arms (Fig. S1). There was no significant correlation between these two immune measurements (Spearman’s rho: −0.05, p-value = 0.72).

The time to onset of parasitaemia in those individuals who became infected was significantly different between the two arms (log rank test: p-value = 0.04 [23]) with the delayed-fractional arm showing longer delays in detectable time to onset of infection (Fig. S2). These results taken together provide evidence for investigating the influence of the antibody immune response characteristics on both the level of protection afforded and also on the influence of reducing parasite load that might lead to delays in the production of merozoite numbers necessary for detection of parasitaemia.

### 3.2 Model fitting

To assess the contribution of anti-NANP antibody titre and avidity to overall vaccine efficacy, we compared the fit of models incorporating either IgG titre data, avidity data, or both titre and avidity from the challenge study using Bayesian methods. The model that provided the best fit to the data included both anti-NANP antibody titre and antibody avidity, with the DIC indicating this to provide a better fit than models with either antibody titre or avidity alone (Table 2 and Table S1). Furthermore, based on the DIC, avidity alone provided a better fit than antibody titre alone. Including an interaction term between titre and avidity to capture synergistic effects did not result in a better fit to the data. We found that the best fitting dose–response curves modelled antibody titre according to the Hill-function and avidity according to the exponential (Table S1 and Fig. S3). The best fitting model parameter values and their descriptions are given in Table 1.

**Table 2:** **Comparison of models where protection from infection depends on antibody titre and antibody avidity, titre or avidity alone and a model including an interaction term.** The ranking of models by DIC highlights the finding that the data is best explained by a model that includes both anti-NANP antibody titre and avidity index without an interaction term.

**Model**	**Difference in DIC compared to best fitting model**
Antibody titre, Avidity	0.000
Avidity only	2.049
Antibody titre, Avidity with an Interaction term	2.069
Antibody titre only	12.57

**Fig. 2:**
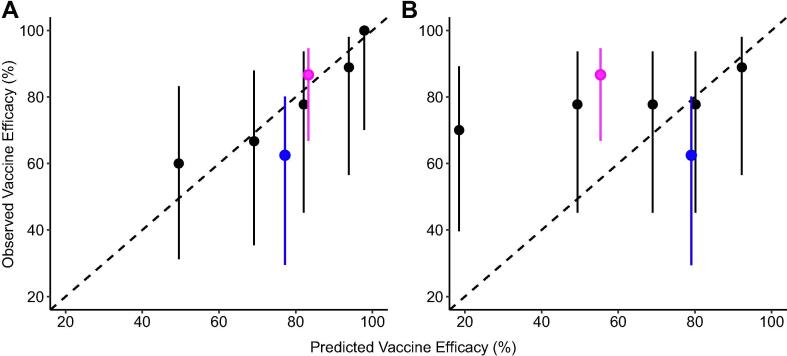
**Comparisons of model predicted vaccine efficacy resulting from the sporozoite-infection model when (A) anti-NANP IgG antibody titre and avidity are included as predictors of vaccine efficacy and (B) when only anti-NANP IgG antibody titre is included as a predictor of vaccine efficacy.** The dashed black line represents equivalent predicted and observed efficacy. Pink points represent the Fx017M volunteers and blue points the 012 M volunteers. Black points represent all trial volunteers grouped into quintiles according to their model predicted vaccine efficacy. Vertical lines represent binomial 95% confidence intervals for observed efficacy in each subgroup. (For interpretation of the references to colour in this figure legend, the reader is referred to the web version of this article.)

Despite the low number of trial participants, we found that the titre-avidity model replicated well the observed vaccine efficacy of individual participants (Fig. 2A, Table 3) and their recorded time to onset of parasitaemia (Fig. S2A). The inclusion of avidity into the sporozoite infection model as a predictor of vaccine efficacy allowed us to better capture the efficacy relationship between immune markers and protection status compared to a model that only took antibody titre into account. The titre only model failed to capture the increase in vaccine efficacy of the delayed-fractional arm over the standard arm and instead overestimated the efficacy from the standard arm and underestimated the efficacy of the delayed fractional arm (Fig. 2B). While it was harder to replicate the delays in the time to onset of parasitaemia we found that the titre only model did not manage to capture the observed delays from the trial. This titre only model predicted earlier onsets of infection for individuals across both arms of the trial, whereas the inclusion of avidity dose–response terms better replicated the observed relationship and distinguished the later times to onset from the delayed-fractional arm when assessed visually (Fig. S2).

**Table 3:** **Comparison of predicted (red) and observed (black) vaccine efficacy against infection at first challenge for the sporozoite infection model**. Number in brackets represent the number of individuals infected in each tercile/total number in each tercile.

	**Anti-NANP Antibody Titres**			
	**Low** 3399–18,512	**Med** 18513–34,139	**High** 34,140–74,710	**All** 3399–74,710
**Antibody Avidity Index**				
**Low** 28–44	41.0%	61.3%	76.6%	62.4%
	66.7% (1/3)	57.1% (3/7)	80.0% (1/5)	66.7% (5/15)
**Med** 45–59	64.9%	85.2%	92.9%	76.8%
	62.5% (3/8)	100% (0/3)	80% (1/5)	75% (4/16)
**High** 60–94	86.4%	96.0%	98.2%	94.1%
	75.0% (1/4)	100% (0/6)	100% (0/5)	93.3% (1/15)
**All** 28–94	65.5%	77.9%	89.8%	**77.7%** (95% CI 64.2–87.2%)
	66.7% (5/15)	81.3% (3/16)	86.7% (2/15)	78.2% (10/46)

### 3.3 Model predicted vaccine efficacy as a function of the antibody response

**Fig. 3:**
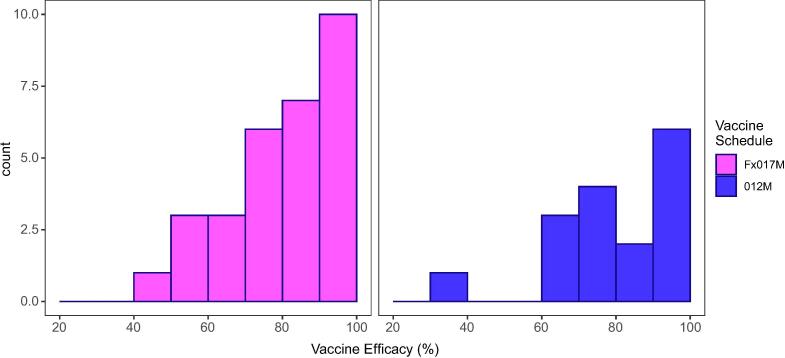
**Distribution of model predicted vaccine efficacy against infection for each volunteer (n46) in the challenge trial by vaccination schedule arm.** This shows there is significant variation in predicted efficacy but with a large proportion of volunteers falling in the higher (>80%) vaccine efficacy. The heterogeneity in individual predicted vaccine efficacy results from the underlying heterogeneity in immune measurements between individuals. As there is no threshold of protection for immune measurements, we see this efficacy distribution of the delayed-fractional regime that is characteristic of a leaky vaccine.

We used the best fitting model parameters to calculate vaccine efficacy as a function of achieved immune responses. Protection against infection was estimated to increase with both increasing anti-NANP antibody titre and avidity. Table 3 shows the observed and model-predicted vaccine efficacy against infection for volunteers stratified into terciles (low, medium, high) based on their titre and avidity measurements. Of the five individuals with the highest recorded immune response measurements, all were protected following challenge. When we estimated vaccine efficacy as a function of these recorded immune responses efficacy was extremely close to sterile protection upon first challenge at 98.2% (95% CrI 91.6–99.7%). If high avidity levels were achieved (index > 60) but antibody titre was in the lowest tercile, vaccine efficacy was estimated to be 86.4% (95% CrI 62.4–96.6%). If high titres were induced but avidity remained low (index < 44) efficacy against infection was estimated to be 76.6% (95% CrI 53.6–92.4%). Lower levels of efficacy were predicted if both induced titre and avidity levels were in the lowest terciles (predicted efficacy 41.0% [95% CrI 22.2–59.2%]). The estimated distribution of individual efficacy against infection (Fig. 3) showed substantial variation. While the delayed-fractional arm showed higher overall efficacy, there remained a large amount of variation between volunteers within this arm when efficacy is predicated at the individual level due to the underlying variation in immune response measurements between individuals.

**Fig. 4:**
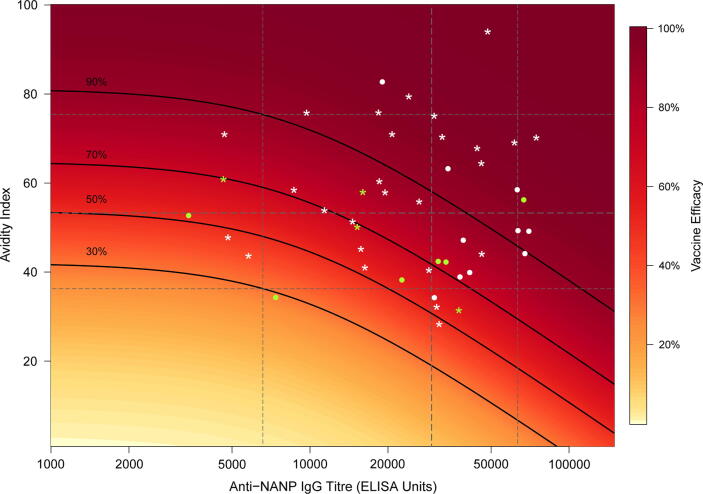
**Predicted efficacy against infection as a function of anti-NANP IgG antibody titre (ELISA Units) and avidity index.** Vertical dashed lines represent the median and 90% ranges of the observed antibody titres. Horizontal dashed lines represent the median and 90% ranges of the observed avidity index measurements. Isoclines represent the 30%, 50%, 70% and 90% estimated vaccine efficacies for combinations of avidity and titre. Green represents infected volunteers and white protected volunteers, * the Fx017M arm and • the 012 M arm. (For interpretation of the references to colour in this figure legend, the reader is referred to the web version of this article.)

**Fig. 5:**
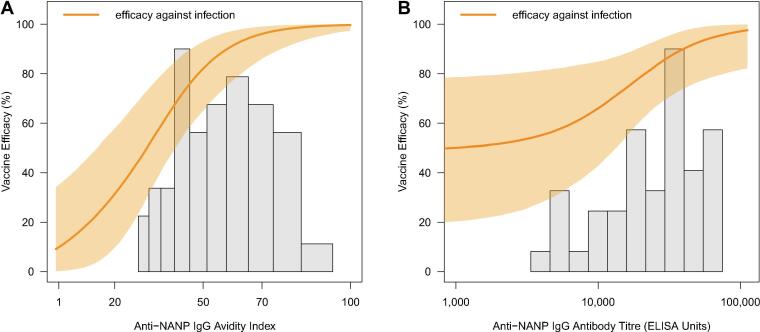
**Predicted vaccine efficacy against infection for (A) increasing IgG avidities predicted at the median levels of IgG antibody titre (ELISA Units) observed in the challenge study and (B) for increasing IgG antibody titre (ELISA Units) predicted at the median IgG avidity measurement from volunteers in the challenge study.** Histogram showing the distribution of avidity indices and titres respectively from vaccinated volunteers across both vaccine schedule arms. Median levels from the study avidity index: 53.5 and titre: 29,459 ELISA Units. The shaded regions represent the 95% prediction intervals of the estimated vaccine efficacy.

Fig. 4 shows the model predicted vaccine efficacy for a continuum of antibody titre and avidity combinations with the points denoting each individuals’ immunological measurements from the challenge study. Increasing avidity was estimated to substantially increase vaccine efficacy for all but the highest antibody titres. From this graph we observe that all infected participants in the study had avidity measurements below 60 (green points Fig. 4), while their titre measurements varied across the observed range. This is further illustrated in Fig. 5 where we observe the efficacy dose–response curves resulting from a continuous increase in titre and avidity alone. The greatest rate of increase in predicted vaccine efficacy occurs up to an avidity index of approximately 60 before the curve begins to plateau at the highest avidity levels. The increase in predicted efficacy per unit increase in antibody titre progresses more slowly, plateauing only towards the upper end of the observed titre measurements. To visualise the effects of continuous increases in each measurement alone the other immunological measurement was held constant at the median value observed in the study (IgG avidity: 53.5, IgG titre: 29,459).

### 3.4 Vaccine efficacy per sporozoite

In addition to estimating vaccine efficacy against infection for a given level of immune response we also estimated the proportional reduction in the number of parasites emerging from the liver as a result of the antibody immune response. We estimated the efficacy per sporozoite across the vaccination arms to be extremely high at 99.6% (95% CrI 98.8–99.8%). This suggests that even if 1% of the infecting sporozoites escape the immune response they can go on to initiate break through infection, all-be-it at a slower rate than in unvaccinated populations.

## 4 Discussion

Understanding the nature of the protective immune response elicited by second-generation RTS,S vaccines and the identification of immunological markers that reliably predict protection against malaria infection is critical for vaccine development and evaluation. Here, by fitting a biologically-motivated mathematical model of sporozoite infection to data from a recent challenge study, we sought to estimate dose–response relationships between antibody immune response markers and protection from malaria infection. We have demonstrated the added predictive value of including IgG avidity indices, measured using inhibition ELISAs, into the model. And model fits demonstrate how the combination of both IgG titre and avidity help to explain the observed efficacy improvements of the delayed-fractional regime better than if titre alone was used to predict efficacy.

Our results suggest that RTS,S induced protection is dependent on both the quantity and quality of the vaccine induced antibody response but that these two characteristics do not provide any synergistic protective effects as shown through the model fitting. This aligns with our biological understanding that concentration and affinity of antibodies rise after vaccination and during infections but vary independently of each other and reflect different biological processes [27], [43], [44]. At the lower levels of avidity a higher antibody titre was required to achieve a given level of vaccine efficacy, suggesting that low functional avidity could be compensated for by high IgG titres and vice versa [33], [45]. One of the continual challenges of pre-erythrocytic vaccine design is the need to eliminate all circulating sporozoites, as a single sporozoite can be enough to cause breakthrough infection. Our results indicate that delayed-fractional RTS,S prevents the majority of sporozoites surviving the pre-erythrocytic phase with an extremely high level of estimated efficacy per sporozoite. Higher avidity antibodies that recognise and strongly bind sporozoites preventing them from initiating liver stage infection might be important in the short time frame following inoculation to successfully clear invading parasites [46], [47], [48]. However, the protective functions of antibodies against pre-erythrocytic malaria stages are still not fully understood [44] and at this stage we do not know whether higher avidity is simply a marker of increased affinity maturation or if it also has a mechanistic role in protection. Further studies are required to understand the potential functional role high avidity antibodies may have in protection as well as further understanding the role of high avidity antibodies as a correlate of vaccine-induced protection.

To achieve the most effective immune response for sporozoite killing the highest combinations of achievable titre and avidity in this challenge study predicted higher levels of efficacy. However, immune characteristics not explicitly captured in the model may also influence the level of protection afforded at the observed levels of our immunological measurements. One aspect of the antibody response not captured here is the subtype composition of IgG antibodies which may have implications for the activation of functional downstream protective processes [43], [44], [49], [50], [51]. While we have not captured the subtype composition in this work, a recent study suggested that the observed avidity improvements might result from an underlying change in the IgG subclass composition, with a dominant IgG4 response in those volunteers from the delayed-fractional regime [52]. Differing subclass compositions have been found to be associated with protection or risk of malaria infection in various studies with vaccination showing differential subclass patterns to naturally acquired immunity [44], [51], [52], [53], [54], [55]. Given that IgG subclass has implications for antibody function it will be important to further understand the implications of subclass ratio in protective immune responses and if total IgG avidity measurements correlate with subclass composition. These findings in addition to the recent work by Pallikkuth et al. [56] suggest that the delayed-fractional schedule has implications for antibody maturation pathways and subclass switching that are potentially driving changes in the measured immune response and improving vaccine efficacy. The increased predictive value of avidity demonstrated in this work warrants further exploration of its combined utility as a correlate of vaccine induced protection and IgG subclass composition along with a deeper understanding of the number of other immune response variables it might likely mirror. It will be important to further characterise therefore whether total IgG avidity indices themselves can act as a successful surrogate marker of both serum antibody composition and protection.

While this challenge study was performed in malaria naïve adults in the United States, the measurements of avidity to repeat region antigens recorded from the standard arm (mean 45.5) reflect those recorded from RTS,S/AS01 vaccinated children in malaria-endemic countries (median 41.2 [36], mean 45.5 [34] and geometric mean 39 [37]). While the approaches and methodologies of the studies vary making comparisons difficult, if the improvements in avidity observed in the delayed-fractional arm translate to target populations in malaria endemic countries we could see further increases in efficacy. To further optimise the protective efficacy of RTS,S it will be important to disentangle the influence of the fractional dose versus the delay on both immunological markers and vaccine efficacy. Such evaluations are currently ongoing in naturally exposed populations (clinicaltrials.gov identifiers: NCT03276962). Our results show that it will be important to measure both antibody markers in these studies to further assess their utility as predictors of protection in naturally exposed populations.

These field trials in non-naïve naturally exposed populations will also enable us to further understand the relationship between these immune markers and protective efficacy over a longer follow up time in a heterologous *P. falciparum* species environment with variable transmission intensities. A current limitation of the predictability of controlled human malaria infection challenge trials is being able to understand the potential of the vaccine to perform in a diverse parasite strain environment, as only a vaccine matched parasite strain is used. It has previously been shown that the genetic diversity of the malaria parasite can influence the efficacy of RTS,S, with mismatch strains showing reduced efficacy in naturally exposed children [56]. As such, the next stages of field trials will be critical to further our understanding of the immunological and efficacy implications of these dose and schedule changes to RTS,S/AS01 and potentially further understand the associative and functional mechanisms underlying protection. An important consideration for field trial immunogenicity evaluations will also be the baseline levels of anti-NANP antibodies in pre-exposed populations and the potential for parasite exposure between vaccine doses which could impact the adaptive immune response [56]. As challenge trial volunteers have been shown previously to have no or low detectable levels of anti-CSP antibodies pre-vaccination [57], [58], [59], [60] and parasite exposure is timed we cannot take these factors into account in this work.

There are several other limitations to this study. Firstly, the dataset and the number of individuals who became infected following challenge (ten volunteers, six standard and four delayed-fractional) was small, which limits the precision of our estimated biological parameters. Given the small study size informative priors were therefore needed to ensure convergence during model fitting. Second, the model itself has been previously validated but carries intrinsic limitations, as outlined by White et al, including that the sporozoite model is a simplification of a complex system, and that the growth rate of blood-stage parasites is assumed to be constant and the same for all study participants [10]. Which might be why the model struggled to capture the longer delays in onset of parasitaemia observed in the trial. Third, we present a simplified view of the adaptive immune response landscape in this study as we do not have cellular immune data and therefore rely on the assumption that the antibody response drives sporozoite killing. It has been shown that antibody responses show the largest associative role with protection [10], [17], [18], [19] and we therefore show singularly the vaccine efficacy predicted for differing levels of titre and avidity. However CD4+ T cell responses may also provide protective capacity and facilitate antibody production [17], [56], [61], had we had cellular data the efficacy predicted by the model may vary as an additional protective relationship is assumed [10]. In addition, we focus here on NANP repeat antibodies, however recent work has highlighted an association between C-terminal avidity but not titre and time to first malaria episode in standard schedule vaccinated children, which remained after adjusting for NANP IgG titre [37]. Further exploration of the C-terminal as a correlate of vaccine-induced protection along with repeat region antibodies is warranted and is ongoing for the delayed-fractional RTS,S schedule. A further limitation and generalisability problem with this current work is that we rely on avidity measurements from inhibition ELISA with only 4 M urea used as the chaotropic regent. The use of this assay has its own limitations and these results therefore might not correlate to avidity indices evaluated from different assays (including thiocyanate or guanidine HCL inhibition ELISA, plasma magnetic resonance, bilayer interferometry or multiplex assays) [62], [63]. Further work is needed to standardise avidity measurements in malaria vaccine research to improve the validity and precision in avidity as a potential correlate of vaccine efficacy. However, the White et al. model [10] is a highly flexible framework which is one of its many strengths such that further immune mechanisms could be built into the evaluation framework in the future when the data from challenge and field trials is available.

Given the challenge study design, we are only able to estimate the association between vaccine induced immune responses and protective efficacy against challenge. The dynamics of these antibody responses over time are also important for understanding the duration of protection afforded by the vaccine. It is clear that the potential public health impacts of the updated delayed-fractional RTS,S regime will be dependent on the duration of protection afforded by the antibody response as has been shown previously for the standard immunization regime [19], [64]. What we do not yet know is whether the potential increase in avidity of antibodies following the updated delayed-fractional regime, will affect their decay dynamics. For example, whether antibodies of lower avidity wane faster than those of high avidity and if avidity itself is sustained at high levels or does this too wane over time. Using insights obtained from the antibody decay dynamics of the standard dosing regimen [19], a recent modelling study predicted the potential population level impact of an RTS,S/AS01 regime with initial efficacy at levels observed for the delayed-fractional regime. Their results suggest that, with an additional fourth booster dose, this regimen could potentially avert 21–25% more clinical cases than the current RTS,S/AS01 dosing regimen across four representative transmission settings [64]. It will be important to further refine these estimates when antibody decay dynamics have been characterised following longer-term field trials with the updated delayed-fractional regime, which are now underway (clinicaltrials.gov identifier: NCT03276962).

## 5 Conclusions

In malaria vaccine development considerable focus has been on the quantity of the vaccine-induced immune response. Here we have shown that the quality of these induced responses is also an important consideration when evaluating associations between immune markers and protection from infection. Given the need for continued development of a highly efficacious malaria vaccine and the challenges of testing new vaccine formulations in large field trials, the establishment of immune correlates will be invaluable. These results provide an early insight into the use of avidity as a surrogate marker of the quality of the vaccine-induced antibody response to form part of the vaccine evaluation framework moving forwards.

## Code and Data Availability

The R code and underlying data used in the analysis is available at: https://github.com/ht1212/quality_quantity_modelling.

## CRediT authorship contribution statement

**Hayley A. Thompson:** Conceptualization, Software, Formal analysis, Investigation, Writing - original draft, Writing - review & editing. **Alexandra B. Hogan:** Conceptualization, Software, Investigation, Writing - review & editing. **Patrick G.T. Walker:** Conceptualization, Software, Investigation, Writing - review & editing. **Michael T. White:** Conceptualization, Methodology, Software, Investigation, Writing - review & editing. **Aubrey J. Cunnington:** Writing - review & editing. **Christian F. Ockenhouse:** Conceptualization, Resources, Writing - review & editing. **Azra C. Ghani:** Conceptualization, Writing - review & editing.

## Declaration of Competing Interest

The authors declare the following financial interests/personal relationships which may be considered as potential competing interests: ‘ACG has a data-transfer agreement with GSK Vaccines to cover ongoing analysis of trial data related to the RTS,S vaccine. She does not receive any funding from GSK for this work. All other authors declare no competing interests.’.
