# Supplementary material for: Modelling the roles of antibody titre and avidity in protection from Plasmodium falciparum malaria infection following RTS,S/AS01 vaccination
Source: Vaccine. 2020 Nov 3;38(47):7498–507. doi: 10.1016/j.vaccine.2020.09.069 (PMC7607256; doi:10.1016/j.vaccine.2020.09.069)
Supplement: Supplementary data 1 [file mmc1.docx]

**Supplementary Information for:**

**Modelling the roles of antibody titre and avidity in protection from Plasmodium falciparum malaria infection following RTS,S/AS01 vaccination**

**Supplementary Methods:**

**Sporozoite Model**

We assumed that, due to the high variation in the number of sporozoites each mosquito inoculates during a blood feed [1-3], the number of sporozoites that complete hepatic development follows a Negative Binomial Distribution with mean ($n)$ and standard deviation $(\sigma_{n})$. The probability that after challenge, $k$ sporozoites will successfully initiate blood stage infection is then given by:

$S_{k}= \left( \begin{matrix} k+r-1 \\ k \end{matrix} \right)\frac{r^{r}n^{k}}{\left( n+r \right)^{r+k}}$,

where *r* is a shape parameter of the Negative Binomial distribution given by the expression:

$r= \frac{n^{2}}{\sigma_{n - n}^{2}}$.

Each sporozoite that survives intrahepatic development will release merozoites into the blood stream $(T_{L}=6.5)$days after challenge [4, 5]. The number of merozoites that are released into the blood stream was assumed to be Gamma-distributed with mean ($\mu)$ and standard deviation $(\sigma_{\mu})$. These merozoites replicate in the blood at a fixed rate $(m)$[6] until they reach the threshold number for detection of infection by microscopy ($P_{T}$)[7] and treatment begins.

If onset of parasitaemia is recorded on day $(T)$, the initial infectious dose of merozoites emerging from the liver can be expressed as $Q=p_{T}m^{-(T-T_{L})}$. Therefore, any delays in the onset of parasitaemia can be used to estimate the reduction in merozoite number emerging from the liver, enabling a comparison of vaccine efficacy in terms of reductions in parasite number to be calculated.

**Antibody Component**

We adapted the model to account for the possibility that the reduction in parasite load and the prevention of infection may depend upon the relative avidity of anti-NANP antibodies, in addition to the overall titre of these antibodies. To model the influence of these characteristics on the probability of sporozoite survival, dose-response curves were incorporated into the sporozoite infection model. These dose-response curves relate the number of anti-sporozoite antibodies and their avidities (the dose) to the observed probability of an individual being protected following challenge (the response). Two parametric dose-response curves were considered when characterising these relationships: exponential and Hill functions. For an exponential dose-response, the probability that a sporozoite survives the immune response $x$ of a given magnitude is ${f\left( x \right)=e}^{-log(2)\frac{x}{\beta}}$. For a Hill function the probability is given by $f\left( x \right)= \frac{1}{1+\left( \frac{x}{\beta} \right)^{\alpha}}$ where $\alpha$ and $\beta$ are the shape and scale parameters of the respective dose-response curves to be estimated during model fitting. Dose-response curves were assessed as providing protection independently of each other by multiplying dose response curves. We also assessed if there were any synergistic or less than multiplicative interactions between the antibody response measures by modelling an interaction dose response curve as explained in White et al using the following function [8, 9]:

$$f \left( x_{ab} , x_{av} \right)= e^{- \frac{log(2)}{2}\left( \left( \frac{x_{ab}}{\beta_{ab}}+\frac{x_{av}}{\beta_{av}} \right)+\sqrt{\left( \frac{x_{ab}}{\beta_{ab}}+\frac{x_{av}}{\beta_{av}} \right)^{2}}+4\gamma\frac{x_{ab}x_{ab}}{\beta_{ab}\beta_{av}} \right)}$$

where $\beta ab/av$ are scale parameters and $\gamma$is a shape parameter, $\gamma$ >0 suggests a synergistic interaction between immune markers, $\gamma$=0 suggests immune markers act independently and -1 < $\gamma$ <0 suggests a less than multiplicative effect of immune markers. Given the vaccine induced antibody immune responses, the mean number of successful sporozoites following challenge will be reduced from $n$ to $nf(x)$, such that we update the sporozoite survival probability by replacing $n$ with $nf(x)$:

$$S_{k}(x)= \left( \begin{matrix} k+r-1 \\ k \end{matrix} \right)\frac{r^{r}{(nf\left( x \right))}^{k}}{\left( nf(x)+r \right)^{r+k}}$$

**Model Likelihood**

Given these models an individual’s probability of infection and expected time to onset of parasitaemia will depend upon several parameters:

- The mean and standard deviation of the number of successful sporozoites $(n, \sigma_{n})$
- The mean and standard deviation of the number of merozoites released per successful sporozoite $(\mu, \sigma_{\mu})$
- The dose-response parameters for the antibody immune mechanisms relating the magnitude of these responses to the probability of sporozoite survival following vaccination $(\alpha,\beta ab\&av)$

Given the results from the challenge study: infection status $(I)$, immune measurements of titre and avidity $(x)$, time to onset of parasitaemia $(T)$ and the resulting estimate of the liver to blood inocula $(Q)$ that above parameters can be estimated using the following likelihood equation:

$$L\left( n, \sigma_{n}, \sigma_{\mu}, \beta_{ab},\alpha_{ab},\beta_{av},\alpha_{av} \right|I, Q, x )= \prod_{j=1}^{j} \left( {{\left( \sum_{k=1}^{\infty} S_{k}\left( x_{j} \right) \left( \frac{\mu}{\sigma_{\mu}^{2}} \right)^{\frac{\mu^{2}}{\sigma_{\mu}^{2}}} \frac{{Q_{j}^{\frac{\mu^{2}}{\sigma_{\mu}^{2}} -1} e}^{-\frac{Q_{j}\mu}{\sigma_{\mu}^{2}}}}{\Gamma\left( \frac{\mu^{2}}{\sigma_{\mu}^{2}} \right)} \right)^{I_{j}} S}_{0}\left( x_{j} \right)}^{1-I_{j}} \right)$$

The likelihood can be explained when taken in its constitute parts. $j$ indexes the number of volunteers in the challenge study and $k$ the number of sporozoites injected. For a volunteer who was protected $(I_{j}=0)$ then $k=0$ sporozoites will be successful with the probability $S_{0}(x_{j})$. If an individual becomes infected $(I_{j}=1)$, then infection will have been initiated by *k* number of sporozoites theoretically from $1$to $\infty$ with the probability $S_{k}(x_{j})$. Each of these successful sporozoites will then release a given number of merozoites into the blood stream following a Gamma distribution $\Gamma\left( \frac{\mu^{2}}{\sigma_{\mu}^{2}},\frac{\sigma_{\mu}^{2}}{\mu^{2}} \right)$.

**Binary Infection Model**

An alternative way to parameterise the sporozoite infection model can be to take into account only the binary outcome of protection status from trial volunteers and ignore the data on the time taken for blood-stage infection to be detectable in infected volunteers. This way the model reduces to a binomial likelihood where the dose-response curves relate directly to vaccine efficacy and not sporozoite survival. After challenge it is thus assumed that volunteer $j( j=1,\ldots,46)$ will either become infected $I=1$ or will be protected $I=0$ depending on the combination of their anti-NANP IgG titre and avidity index $x_{j}$. The likelihood equation for the parameters of the dose-response curves $(\alpha,\beta ab\&av)$ can be written as:

$$L \left( \beta_{ab},\alpha_{ab},\beta_{av},\alpha_{av} \right| I, x)= \prod_{j=1}^{J} {f(x_{j})}^{I_{j}} {(1-f\left( x_{j} \right))}^{1-I_{j}}$$

The results of fitting this model can be seen in Table S3.

The efficacy against infection estimated by the binary infection model is *VE* = 78.1% (95% CI, 76.5% - 79.9%). In the binary model of infection the dose-response curves relate directly to protection from infection and not the proportion of sporozoites killed and therefore can only be used to estimate vaccine efficacy against infection and not per-sporozoite.

**Fig. S1. (A) Box and whisker plots of the observed differences in anti-NANP antibody titre (ELISA Units) from volunteers across both arms of the challenge trial.** Titre measurements are stratified by vaccine schedule and by infection status. Mean measurements for each group are shown by the filled triangles and outliers by grey circles. There were significantly higher antibody titres measured in the standard arm (n 16) compared to the delayed-fractional arm (n 30), Mann-Whitney test comparing all participants in both arms, p-value=0.02. **(B) Box and whisker plots of the observed differences in anti-NANP avidity index from volunteers across both arms of the challenge trial.** Avidity index measurements are stratified by vaccine schedule and by infection status. There was a significant difference in avidity index between the delayed-fractional (n 30) and standard arms (n 16) Mann-Whitney comparing all participants in both arms, p-value=0.03. Mean measurements for each group are shown by the filled triangles and outliers with grey circles.


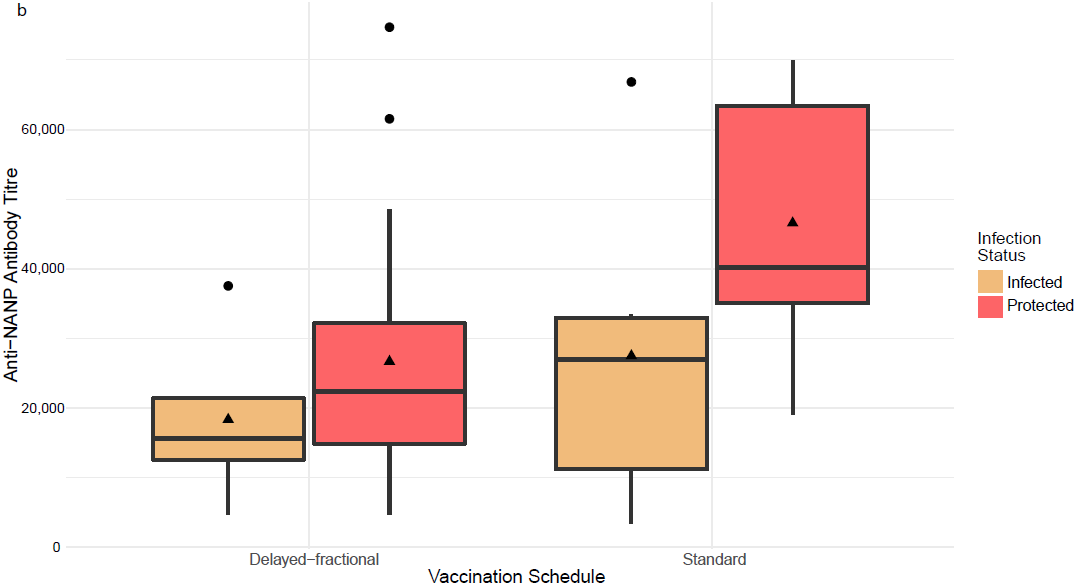


**A**


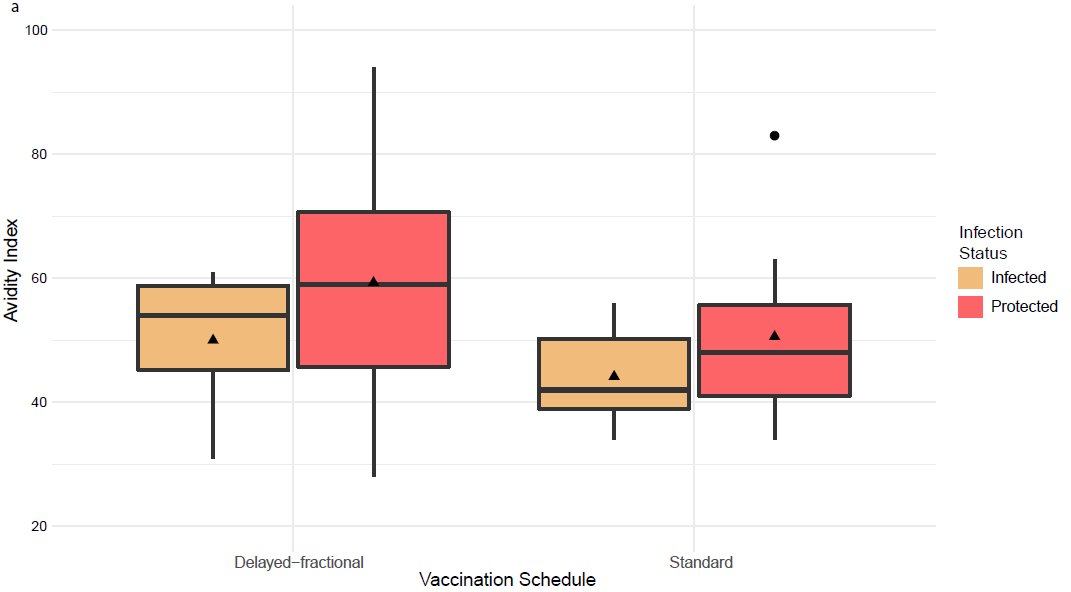


**B**


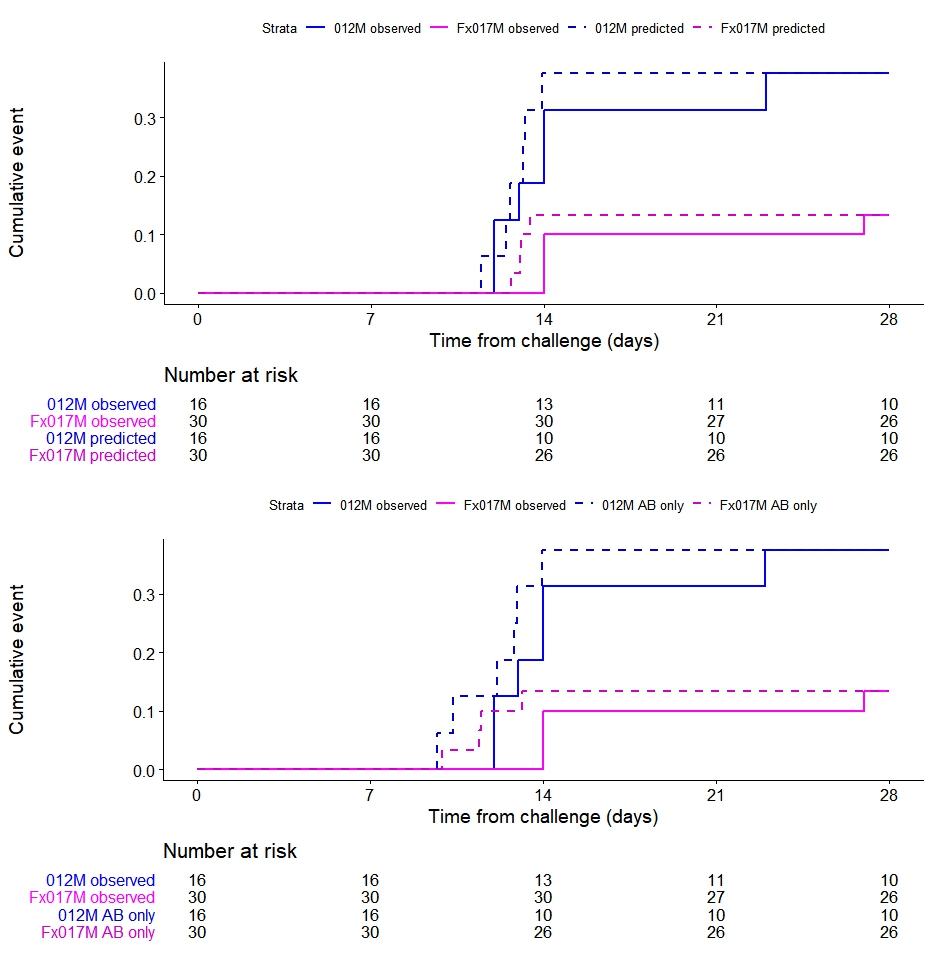


**A**

**B**

**Fig. S2.** **Cumulative incidence of *P. falciparum* parasitemia as observed in each vaccine trial arm (solid lines) and predicted by the model (dashed lines).** Follow up period for the challenge study reached 28 days after which point volunteers are deemed protected from infection following challenge. Infection was determined by a positive blood slide. Thirty subjects received the Fx017M regime and 16 the standard 012M regime, of these 26 and 10 individuals were protected from infection respectively. The Dashed lines in **(A)** represents the antibody titre and avidity model predicted time to onset given the combination of antibody immune responses for each infected volunteer. The model predicts time to onset relatively well but doesn’t replicate the outliers with extreme delay in onset (individuals at day 23 and 27). The dashed lines in **(B)** represents estimated time to onset calculated from the parameters of the antibody only model. This model predicts an earlier onset of parasitaemia for volunteers in both arms and again fails to capture the extreme delays.


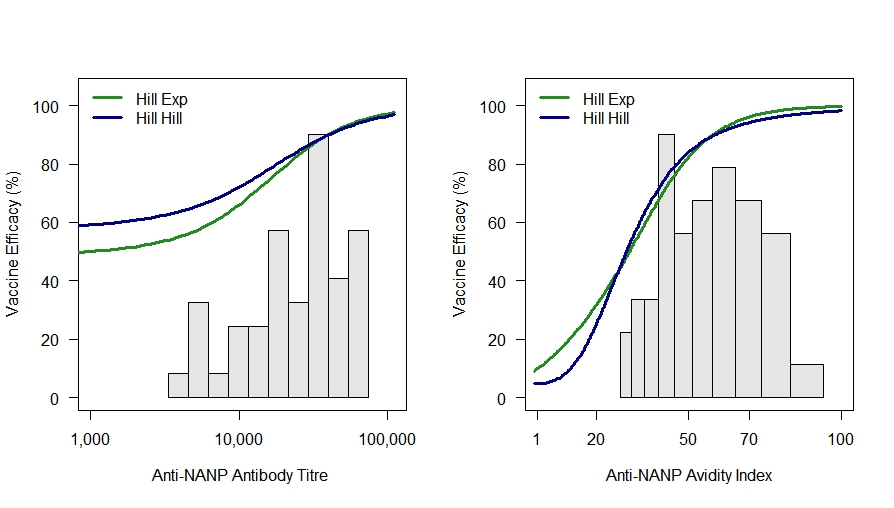


**Fig. S3.** **Model predicted vaccine efficacy against infection for increasing antibody titre and avidity measurements comparing the two best-fitting sporozoite infection models.** Hill-Exp represents a model whereby antibody titre follows a Hill-function dose-response curve and avidity follow an exponential dose-response curve and Hill-Hill denotes a model in which both antibody titre (ELISA Units) and avidity follow Hill function dose-response curves. There is a minimal difference in the DIC (0.6) of these two fitted models and the relationship they predict between titre and avidity despite the functional form differences is highly correlated.

**Table S1. MCMC parameter estimates for sporozoite infection models with different combinations of dose-response curves and model comparisons using Deviance-Information Criterion (DIC).** Hill denotes a Hill-function dose-response curve and Exp and exponential dose-response curve.

| Dose-response curves | | Sporozoite Infection Model Parameters | | | | | | | | | | |
| --- | --- | --- | --- | --- | --- | --- | --- | --- | --- | --- | --- | --- |
| **Titre** | **Avidity** | **n** | **σ_n_** | **σ_µ_** | **β_ab_** | **α_ab_** | **β_av_** | **α_av_** | **γ** | **VE** | **ΔDIC** |  |
| Hill | Exp | 134 | 177 | 71428 | 8636 | 1.53 | 7.92 |  |  |  | 0.000 |  |
| Hill | Hill | 150 | 177 | 70717 | 9560 | 1.31 | 11.70 | 3.42 |  |  | 0.606 |  |
| Exp | Exp | 133 | 179 | 72036 | 11418 |  | 7.80 |  |  |  | 0.767 |  |
| Exp | Hill | 153 | 177 | 70653 | 13027 |  | 11.56 | 3.47 |  |  | 1.056 |  |
|  | Hill | 140 | 184 | 70157 |  |  | 9.38 | 3.75 |  |  | 2.049 |  |
| Interaction | | 132 | 182 | 72112 | 13606 |  | 8.77 |  | 1.08 |  | 2.069 |  |
|  | Exp | 113 | 192 | 72319 |  |  | 6.11 |  |  |  | 3.408 |  |
| Baseline | | 71 | 187 | 74752 |  |  |  |  |  | 0.98 | 8.128 |  |
| Hill |  | 82 | 221 | 75249 | 4308 | 2.76 |  |  |  |  | 12.57 |  |
| Exp |  | 66 | 225 | 76749 | 6019 |  |  |  |  |  | 18.46 |  |

Table S2. Prior distributions used in the MCMC fitting of the sporozoite infection model. Gamma priors were chosen as we assume a multivariate normal posterior distribution of our parameters. Previous evidence for the dose-response parameters was taken from earlier maximum likelihood estimations (MLE) of model fitting and previous data, a uniform distribution was chosen for the shape parameter of the antibody dose response as the parameter was previously characterized for antibody titre data from earlier ELISA tests (different units) therefore a uniform prior was selected with reasonable bounds to allow identification of this parameter for this dataset.

| **Parameter** | **Parameter Description** | **Prior Distribution** | **Previous Data** | **Gamma Shape and Rate Parameters** | **References** |
| --- | --- | --- | --- | --- | --- |
| **n** | Mean number of successful sporozoites per challenge | Gamma | 150 (75, 237) | 7.5, 0.05 | [5, 8] |
| **σn** | Standard deviation of the number of sporozoites per challenge | Gamma | 194 (93, 324) | 9.7, 0.05 | [5] |
| **σµ** | Standard deviation in the number of merozoites per sporozoite | Gamma | Co-efficient of variation 2.09 (1.59 – 3.56)  Standard deviation  62700 (47700, 106800) | 31.35, 0.0005 | [5] |
| **βab** | Anti-NANP antibody titre needed for 50% reduction in sporozoite survival probability | Gamma | MLE estimate ~ 15,000 | 5, 0.0005 |  |
| **αab** | Shape parameter for antibody dose-response | Uniform | 1.32 (0.85-1.77) | Uniform dist (0,30) | [8] |
| **βav** | Antibody avidity index needed for 50% reduction in sporozoite survival probability | Gamma | MLE estimate ~8 | 6, 0.05 |  |

Table S3: Maximum likelihood parameter estimates for binary infection models with different combinations of dose-response curves and model comparison with Deviance-Information Criterion (DIC).

| **Dose-response curves** | | **β_ab_** | **α_ab_** | **β_av_** | **α_av_** | **γ** | **ΔDIC** |
| --- | --- | --- | --- | --- | --- | --- | --- |
| **Titre** | **Avidity** |  |  |  |  |  |  |
| Hill | Hill | 9528 | 0.069 | 12.4 | 0.132 |  | 0.00 |
| Hill | Exp | 546 | 0.165 | 82.5 |  |  | 11.71 |
| Exp | Hill | 94451 |  | 3.4 | 0.359 |  | 14.65 |
| Exp | Exp | 97258 |  | 28.6 |  |  | 90.49 |
| Exp-Exp Interaction | | 93485 |  | 33.4 |  | 1.19 | 95.45 |

**References**

[1] Vanderberg JP. Plasmodium berghei: quantisation of sporozoites injected by mosquitoes feeding on a rodent host. Experimental parasitology. 1977;42:169-81.

[2] Churcher TS, Sinden RE, Edwards NJ, Poulton ID, Rampling TW, Brock PM, et al. Probability of transmission of malaria from mosquito to human is regulated by mosquito parasite density in naïve and vaccinated hosts. PLoS pathogens. 2017;13:e1006108.

[3] Beier JC, Davis JR, Vaughan JA, Noden BH, Beier MS. Quantitation of Plasmodium falciparum sporozoites transmitted in vitro by experimentally infected Anopheles gambiae and Anopheles stephensi. The American journal of tropical medicine and hygiene. 1991;44:564-70.

[4] Murphy J, Baqar S, Davis J, Herrington D, Clyde D. Evidence for a 6.5-day minimum exoerythrocytic cycle for Plasmodium falciparum in humans and confirmation that immunization with a synthetic peptide representative of a region of the circumsporozoite protein retards infection. Journal of Clinical Microbiology. 1989;27:1434-7.

[5] Coffeng LE, Hermsen CC, Sauerwein RW, de Vlas SJ. The power of malaria vaccine trials using controlled human malaria infection. PLoS computational biology. 2017;13:e1005255.

[6] Bejon P, Andrews L, Andersen RF, Dunachie S, Webster D, Walther M, et al. Calculation of liver-to-blood inocula, parasite growth rates, and preerythrocytic vaccine efficacy, from serial quantitative polymerase chain reaction studies of volunteers challenged with malaria sporozoites. The Journal of infectious diseases. 2005;191:619-26.

[7] Bejon P, Andrews L, Hunt-Cooke A, Sanderson F, Gilbert SC, Hill AV. Thick blood film examination for Plasmodium falciparum malaria has reduced sensitivity and underestimates parasite density. Malaria Journal. 2006;5:104.

[8] White MT, Bejon P, Olotu A, Griffin JT, Riley EM, Kester KE, et al. The relationship between RTS, S vaccine-induced antibodies, CD4+ T cell responses and protection against Plasmodium falciparum infection. PLOS one. 2013;8:e61395.

[9] White MT, Griffin JT, Riley EM, Drakeley CJ, Moorman AM, Sumba PO, et al. Efficacy model for antibody-mediated pre-erythrocytic malaria vaccines. Proceedings of the Royal Society of London B: Biological Sciences. 2010:rspb20101697.
